# Clinical Relevance of Antimicrobial Susceptibility Testing Methods in Carbapenem-Resistant *Acinetobacter baumannii* Pneumonia: A Secondary Analysis of a Randomized Controlled Trial

**DOI:** 10.3390/antibiotics15020189

**Published:** 2026-02-09

**Authors:** Chutchawan Ungthammakhun, Vasin Vasikasin, Nadia Cheh-Oh, Wichai Santimaleeworagun, Dhitiwat Changpradub

**Affiliations:** 1 Division of Infectious Disease, Department of Medicine, Phramongkutklao College of Medicine, Phramongkutklao Hospital, Bangkok 10400, Thailand; rebornhue@gmail.com (C.U.);; 2 Department of Clinical Pharmacy, Faculty of Pharmaceutical Sciences, Prince of Songkla University, Songkhla 90110, Thailand; 3 Department of Pharmaceutical Care, Faculty of Pharmacy, Silpakorn University, Nakorn Pathom 73000, Thailand

**Keywords:** carbapenem-resistant *Acinetobacter baumannii*, antimicrobial susceptibility testing, BMD, sulbactam, mortality rate

## Abstract

**Background/Objective**: Carbapenem-resistant *Acinetobacter baumannii* (CRAB) pneumonia has limited treatment options, and sulbactam MIC interpretation varies with the antimicrobial susceptibility testing (AST) method. This study compared sulbactam MICs determined using broth microdilution (BMD) and the E-test and examined their associations with 28-day mortality. **Methods**: This secondary analysis used data from a randomized controlled trial comparing colistin plus sulbactam at 9 g/day versus 12 g/day in adults with CRAB pneumonia. The sulbactam MICs of 134 isolates were determined using BMD and the E-test. The agreement between methods across MIC ranges and associations between MICs, dosing, and 28-day mortality were analyzed. **Results**: Sulbactam MICs determined using BMD were lower than those obtained with the E-test (MIC50/90: 32/128 µg/mL vs. 96/≥256 µg/mL). Overall, agreement between methods was limited and depended on MIC level, with better agreement at lower MICs and marked discrepancies at higher MICs, where the E-test frequently overestimated the MICs. Using the IDSA breakpoint (MIC ≤ 4 µg/mL), susceptibility was identified in 6% of isolates with BMD and 3% with the E-test. A significant survival benefit with high-dose sulbactam (12 g/day) was observed in patients with BMD-determined MICs ≥ 128 µg/mL (HR 0.27; 95% CI, 0.077–0.956; *p* = 0.042), whereas no mortality association was seen when MICs were categorized using the E-test results. **Conclusions**: AST method selection substantially affects sulbactam MIC interpretation in CRAB pneumonia. BMD shows stronger correlation with clinical outcomes than the E-test, particularly at high MIC levels. High-dose sulbactam may benefit patients with highly resistant isolates, underscoring the need for accurate and standardized AST methods.

## 1. Introduction

Carbapenem-resistant *Acinetobacter baumannii* (CRAB) represents a formidable global health threat. It has been identified by both the World Health Organization (WHO) and the Centers for Disease Control and Prevention (CDC) as a top-priority pathogen requiring urgent antimicrobial development efforts [1,2]. This multidrug-resistant (MDR) organism is a leading cause of severe healthcare-associated infections, particularly ventilator-associated pneumonia (VAP) in intensive care units (ICUs) [3,4,5]. The clinical burden of CRAB pneumonia is substantial, with incidence rates reaching up to 649 cases per 1000 ICU patients in southeast Asia [4], and reported mortality rates ranging from 14% to 73% [6,7,8].

Despite its clinical importance, effective therapeutic options for CRAB remain limited. Although novel agents such as sulbactam–durlobactam have shown promise, access to these treatments is still restricted in many parts of the world [9,10]. As a result, sulbactam, a β-lactamase inhibitor with intrinsic bactericidal activity against *A. baumannii* via its binding to penicillin-binding proteins (PBPs) 1 and 3 [11,12], continues to play a central role in treatment. It is often administered in combination with colistin or other antimicrobials [13,14,15]. According to the 2025 Clinical and Laboratory Standards Institute (CLSI) breakpoints, susceptibility for ampicillin–sulbactam is defined as ≤8/4 µg/mL and resistance as ≥32/16 µg/mL, while for sulbactam–durlobactam, susceptibility is defined as ≤4/4 µg/mL and resistance as ≥16/4 µg/mL [16]. Antimicrobial surveillance data from Thailand reveal that over 75% of *A. baumannii* isolates are resistant to ampicillin–sulbactam [17]. Nevertheless, accumulating evidence suggests that high-dose sulbactam regimens (≥9 g/day) are associated with improved clinical outcomes in patients with CRAB-related hospital-acquired pneumonia (HAP) or VAP [10,13,18,19,20,21].

Optimizing sulbactam therapy relies on the accurate determination of its minimum inhibitory concentration (MIC). However, antimicrobial susceptibility testing (AST) for sulbactam poses significant challenges. Various AST methods including broth microdilution (BMD), disk diffusion, gradient diffusion methods (such as the Epsilometer test or E-test), and automated systems are employed in clinical microbiology laboratories to assess bacterial susceptibility [16,22,23]. Among these, BMD is widely regarded as the reference standard due to its precision and reproducibility [16,23], whereas methods like the E-test are frequently used due to their convenience. Nevertheless, concerns remain regarding their tendency to overestimate or underestimate MIC values, particularly near critical breakpoints, and the extent to which they correlate with clinical outcomes [22,24,25,26,27]. Although the Infectious Diseases Society of America (IDSA) and CLSI recommend BMD with a sulbactam MIC breakpoint of ≤4 μg/mL [9,16], the clinical relevance of this threshold has not been rigorously validated in large, well-characterized prospective cohorts.

The variability in sulbactam MICs across different AST methods can lead to discordant susceptibility interpretations, potentially resulting in misclassification of resistant strains and the selection of suboptimal antimicrobial therapy [26,27,28,29]. Furthermore, there is a notable lack of studies directly correlating the in vitro MIC values obtained using different AST methods with meaningful clinical outcomes, particularly in resource-limited settings where BMD is not routinely available. Previous investigations involving other difficult-to-treat pathogens, such as tigecycline-resistant *A. baumannii*, have revealed significant discrepancies and high error rates between the E-test and BMD, raising concerns about the reliability of the E-test as a substitute for the reference method [26,30,31,32]. Similar findings have been reported for *Acinetobacter* spp., where E-test results may erroneously indicate resistance in strains found to be susceptible using BMD [33]. These gaps highlight the urgent need to validate AST methods using clinically relevant endpoints to ensure appropriate treatment selection and improved patient outcomes.

This secondary analysis of a randomized controlled trial comparing sulbactam doses of 9 g/day and 12 g/day in patients with CRAB pneumonia was conducted to evaluate the clinical relevance of sulbactam antimicrobial susceptibility testing. Specifically, this study aimed to examine how differences in MIC determination between the E-test and BMD influence MIC distribution, method agreement, and their association with 28-day mortality. By integrating laboratory susceptibility data with clinical outcomes, this study seeks to clarify the role of AST methodology in guiding effective sulbactam therapy for CRAB pneumonia.

## 2. Objectives

This study aimed to evaluate the clinical relevance of sulbactam antimicrobial susceptibility testing in the treatment of CRAB pneumonia by examining the differences in MIC distributions and agreement between the E-test and broth microdilution methods and by assessing how the MIC values derived from each method are associated with 28-day mortality.

## 3. Results

### 3.1. Minimum Inhibitory Concentration (MIC) Distributions

**Figure 1:**
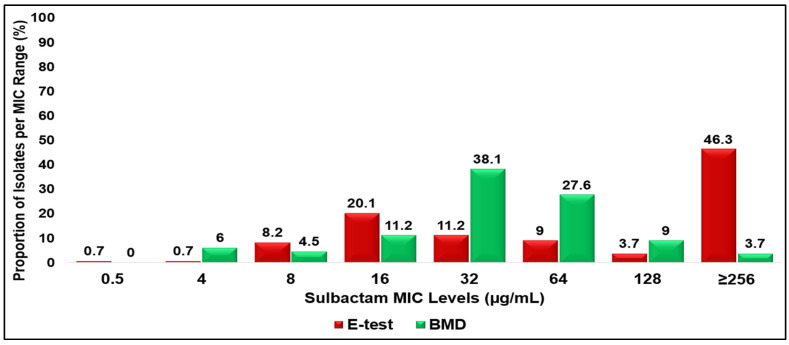
Distribution of sulbactam MICs among CRAB isolates as determined by E-test and BMD methods.

A total of 134 clinical isolates of CRAB—68 from the 9 g/day group and 66 from the 12 g/day group—were evaluated for sulbactam susceptibility using BMD methods as the reference methods and compared with the E-test. The distribution of sulbactam MICs among CRAB isolates varied between the E-test and BMD methods. Using the E-test, the majority of the isolates exhibited high MIC values, with an MIC_50_ of 128 µg/mL and an MIC_90_ of ≥256 µg/mL. Only 1.4% of the isolates were categorized as susceptible (MICs ≤ 4 µg/mL), while 90.3% were classified as resistant (MICs ≥ 16 µg/mL). In contrast, the BMD method demonstrated a lower MIC distribution, with an MIC_50_ of 32 µg/mL and an MIC_90_ of 128 µg/mL. According to this method, 6.0% of the isolates were susceptible to sulbactam, and 89.6% were resistant. Overall, the E-test tended to yield higher MIC values than the BMD method, resulting in more isolates being classified as highly resistant (Figure 1).

### 3.2. Correlation of Sulbactam MICs by E-Test and Broth Microdilution

With the E-test, 22 isolates demonstrated identical MIC values to those obtained with BMD, while 43 isolates showed MICs within ±1 log_2_ dilution. The remaining 69 isolates exhibited discrepancies of ±2 log_2_ dilutions, with both higher and lower MICs obtained with E-test compared with BMD. Overall, the E-test method achieved an EA of 49% across all tested isolates. Further analysis showed that when only analyzing isolates with MICs of ≤64, ≤32, and ≤16 μg/mL, the E-test method showed EA values that were of 55, 69, and 79% higher than those of BMD, respectively (Table 1).

**Figure 2:**
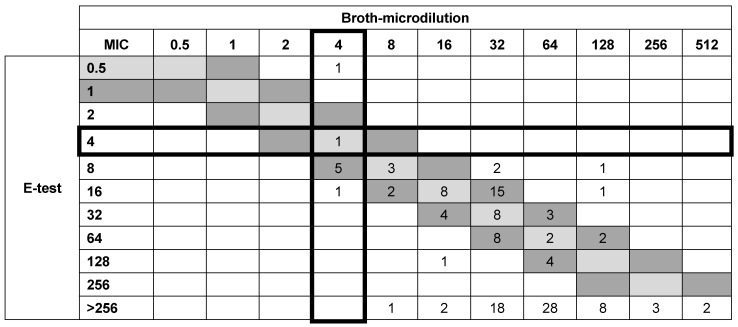
Scattergrams showing numbers of isolates (n = 134) with sulbactam minimal inhibitory concentration determined via agar dilution, E-test versus broth microdilution as a reference method. Solid lines represent the IDSA-recommended susceptibility breakpoint of sulbactam with an MIC ≤ 4 μg/mL. The diagonal boxes (light gray and dark gray) indicate categorical agreement and essential agreement.

For the IDSA-recommended susceptibility breakpoint of sulbactam with an MIC ≤ 4 μg/mL, the E-test method showed a CA of 95.5% (128/134) compared with BMD. The false-susceptible or VME rate of the E-test was 0% (0/126), while the false-resistant or ME rate was 75% (6/8) (Figure 2).

**Figure 3:**
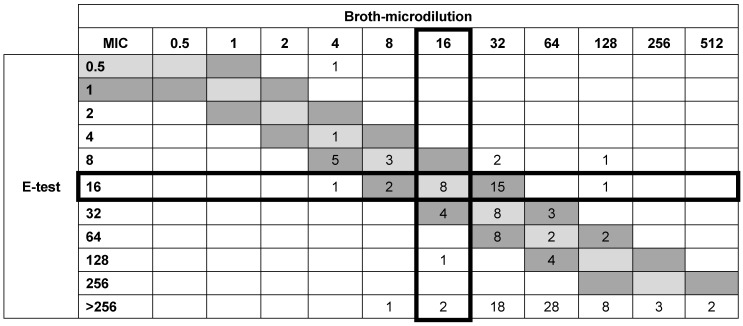
Scattergrams showing numbers of isolates (n = 134), with sulbactam minimal inhibitory concentration determined by agar dilution, E-test versus broth microdilution as a reference method. Solid lines represent MIC of sulbactam with MIC ≤ 16 μg/mL. The diagonal boxes (light gray and dark gray) indicate categorical agreement and essential agreement.

Using sulbactam with an MIC ≤ 16 μg/mL, the E-test demonstrated a CA of 80% (107/134) compared with BMD. With this cutoff, major errors (false-susceptible results) were observed in 18% (19/105) of the isolates, while major errors (false-resistant results) occurred in 28% (8/29) (Figure 3).

### 3.3. Subgroup Survival Analysis by MIC Levels

To explore the association between sulbactam MIC, dosing regimens, and patient outcomes, we conducted survival analyses stratified by sulbactam dose (9 g/day vs. 12 g/day) and MIC values determined by both methods. Survival probability was assessed over 28 days.

**Figure 4:**
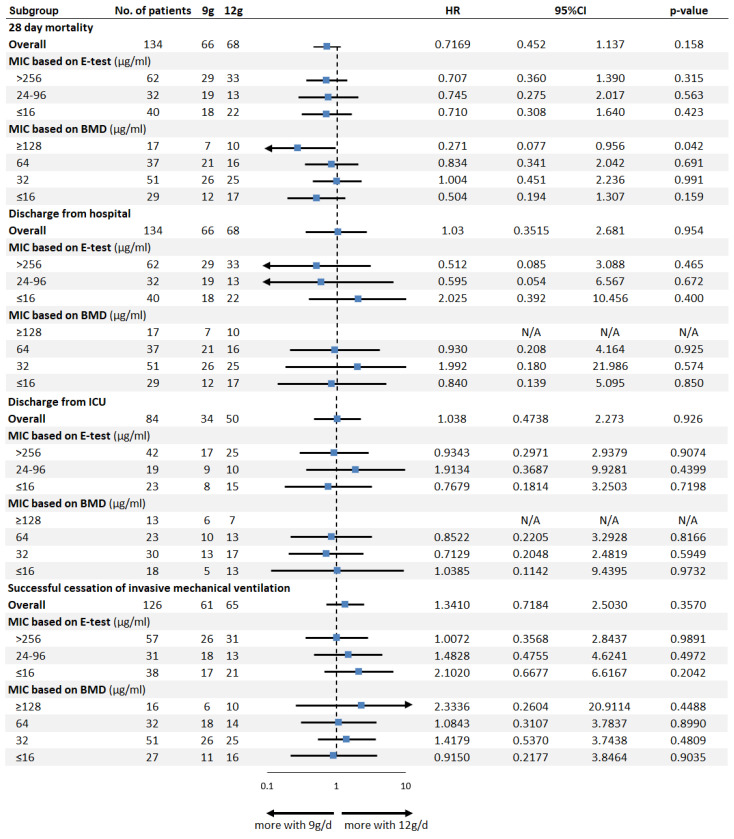
Forest plot of subgroup analyses of different clinical outcomes by sulbactam MIC values, determined using either the E-test or broth microdilution (BMD) method.

The subgroup analyses of clinical outcomes by sulbactam MIC values are shown in Figure 4. The overall hazard ratio (HR) favored 12 g/day over 9 g/day dosing (HR: 0.717; 95% CI: 0.452–1.137; *p* = 0.158 by log-rank test). No statistically significant differences were found between the two treatment groups for either discharge from hospital and ICU alive, or successful cessation of invasive mechanical ventilation.

Among patients with sulbactam MIC ≥ 128 µg/mL determined with the BMD method, high-dose sulbactam (12 g/day) was associated with significantly lower 28-day mortality than 9 g/day (HR: 0.271; 95% CI: 0.077–0.956; *p* = 0.042 per log-rank test). No significant differences in mortality were observed between dosing regimens across other MIC subgroups or in any MIC determined by the E-test method (Figure 4).

**Figure 5:**
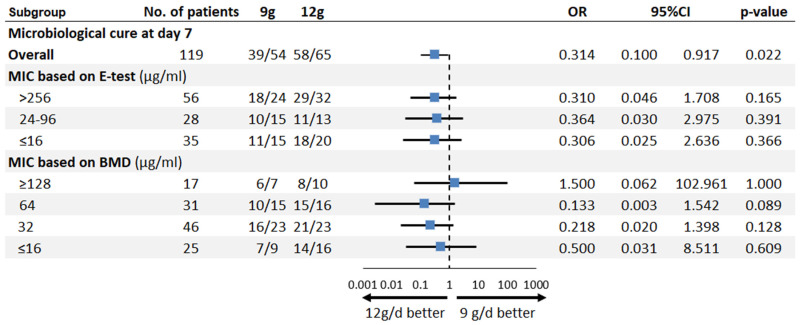
Forest plot of subgroup analyses of microbiological cure by sulbactam MIC value, determined using either the E-test or broth microdilution (BMD) method.

For microbiological cure on day 7, a higher rate was observed in the 12 g/day group compared to the 9 g/day group (OR: 0.314; 95% CI 0.100–0.917; *p* = 0.021) (Figure 5).

**Figure 6:**
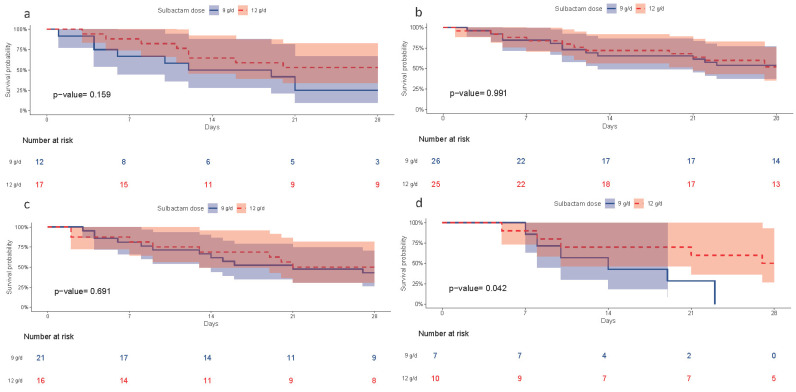
Kaplan–Meier survival curves for 28-day mortality among patients with CRAB pneumonia, stratified according to sulbactam MIC values (as determined by the BMD method) and treatment dosage. Survival outcomes are shown for (**a**) MIC < 32 µg/mL, (**b**) MIC 32 µg/mL, (**c**) MIC 64 µg/mL, and (**d**) MIC ≥ 128 µg/mL.

Among the patients infected with CRAB isolates exhibiting sulbactam MIC < 32 µg/mL, 32 µg/mL, or 64 µg/mL (Figure 6a–c), no statistically significant difference in 28-day survival was observed between those receiving 12 g/day versus 9 g/day of sulbactam. In contrast, a significant survival benefit was observed in the subgroup with MIC ≥ 128 µg/mL (Figure 6d).

## 4. Discussion

In this study, sulbactam MICs among CRAB isolates tended to be high, especially when measured with the E-test. When MICs determined with BMD were compared with previous reports from other regions and time periods, we observed a shift toward higher MIC_90_ values, increasing from 64 µg/mL in earlier studies [34,35] to 128 µg/mL in our cohort. This increase likely reflects ongoing antibiotic pressure, the spread of resistant strains, and regional differences in antibiotic use. When the non-susceptibility cutoff of an MIC ≥ 16 µg/mL was applied, as recommended by the IDSA and CLSI [10,23], a large proportion of isolates were classified as resistant. This finding suggests that standard sulbactam dosing may be insufficient in settings where high MICs are common and highlights the need for continued local resistance surveillance [10,20]. Importantly, the markedly higher MIC values observed with the E-test than with BMD in our cohort may not solely reflect biological resistance. Gradient diffusion methods are known to overestimate β-lactam MICs at very high concentrations due to diffusion limitations [27,33,36], trailing endpoints, and reading difficulties when the inhibition ellipse exceeds the upper range of the strip. Therefore, the apparent shift toward extreme resistance according to the E-test should be interpreted cautiously.

The comparison of MIC testing methods showed limited agreement between the E-test and BMD. Overall, essential agreement was low (49%), and the E-test generally produced higher MIC values than BMD, particularly at higher MIC levels. Almost half of the isolates differed by at least 2 log_2_ dilutions. Agreement improved when only isolates with lower MICs were analyzed, reaching 55%, 69%, and 79% for MICs ≤ 64, ≤32, and ≤16 µg/mL, respectively, indicating better E-test performance at lower MIC ranges. At the IDSA-recommended breakpoint of an MIC ≤ 4 µg/mL, the E-test showed high categorical agreement with no false-susceptible results, but false-resistant results remained common. These differences are consistent with previous studies and are likely related to sulbactam instability and variable drug diffusion in agar-based testing [22,23,24,27,33,36,37]. At very high MIC values (≥128 µg/mL), most E-test results clustered at the upper end of the scale (≥256 µg/mL), limiting their discriminatory ability across resistance strata. In contrast, BMD provided a wider MIC distribution, allowing clearer separation of isolates into clinically meaningful categories.

The choice of MIC testing method has clear clinical implications. The overestimation of MICs of the E-test may lead clinicians to avoid sulbactam and switch to alternative drugs that may be less effective, more toxic, or more expensive. Such misclassification can also affect antimicrobial stewardship efforts and distort local resistance data. On the other hand, the underestimation of MICs could encourage the use of ineffective treatments. In practice, the selection of susceptibility testing methods should consider local MIC patterns and laboratory capacity. In settings where sulbactam MICs are generally low and BMD is not available, the E-test may be used for initial assessments. However, in areas with a high frequency of elevated MICs, BMD should be preferred to ensure accurate MIC results and appropriate treatment decisions for CRAB infection [33].

When patient outcomes were analyzed, the sulbactam MICs measured using BMD were clearly associated with 28-day mortality. Patients infected with isolates showing very high MICs (≥128 µg/mL) had better survival when treated with high-dose sulbactam (12 g/day) compared with standard dosing (9 g/day), suggesting a dose–response relationship in this small subgroup [13,38]. In contrast, increasing the dose did not improve outcomes in patients with lower MIC isolates, indicating that higher doses may not be needed when sulbactam activity is relatively preserved. These findings suggest that high MIC thresholds help identify patients most likely to benefit from high-dose therapy [10,13,19,20]. However, this analysis was post hoc, included a limited number of patients (n = 17), and was not pre-specified in the original trial. Therefore, this finding should be considered hypothesis-generating and interpreted with caution, as it may be influenced by multiple comparisons and residual confounding. Notably, no corresponding survival signal was observed when MICs were stratified using E-test. This discrepancy likely reflects the limited reliability of gradient diffusion methods at very high MIC levels, where most isolates are compressed into the highest category [30,33]. As a result, the E-test may fail to capture biologically relevant differences in sulbactam activity that are better resolved with BMD.

Regarding the microbiological cure rate, there was a statistically significant advantage for the 12 g/day regimen. Interestingly, while the point estimates for nearly all MIC subgroups favored the 12 g/day dose, the benefit was numerically pronounced across higher MICs measured with BMD.

In contrast to the mortality and microbiological outcomes, other indicators of physical recovery—such as ICU discharge and successful discontinuation of mechanical ventilation—exhibited substantially greater variability. This likely reflects the complex interplay between antibiotic effects and host responses, particularly in critically ill patients with high mortality rates and a limited number of survivors discharged from the hospital or ICU within 28 days.

Overall, our findings support the use of MIC-guided therapy, in which dosing decisions are based on accurate MIC values rather than susceptibility categories alone. Precise MIC measurement is important for optimizing treatment, as unnecessary high-dose therapy may increase toxicity without improving outcomes in patients with lower MIC isolates. Although pharmacokinetic and pharmacodynamic data were not directly measured, our results are consistent with β-lactam principles, especially the importance of maintaining drug levels above the MIC for sufficient time (fT > MIC) to achieve optimal antibacterial activity [19,39,40].

This study is strengthened by its randomized controlled trial design and by the direct comparison of MICs obtained with BMD and the E-test in the same patient cohort. The limitations include small sample sizes in some MIC groups, retrospective MIC testing without blinding, lack of pharmacokinetic data, and a single-center setting, which may limit generalizability. Despite these limitations, the consistent results across analyses support the reliability of our conclusions. Future multicenter studies that combine MIC data with pharmacokinetic/pharmacodynamic analysis are needed to refine MIC-guided dosing strategies and to reconsider sulbactam breakpoints for *A. baumannii*.

## 5. Materials and Methods

### 5.1. Study Design and Participants

This study is a secondary analysis of a randomized controlled trial conducted at Phramongkutklao Hospital, Bangkok, Thailand, between September 2019 and September 2023. The original trial compared two sulbactam dosing regimens (9 g/day versus 12 g/day) in combination with colistin for the treatment of CRAB pneumonia [21]. The first CRAB isolated from each patient was used for AST.

### 5.2. Collection of Isolates and Identification

Isolates were collected from sputum specimens and transported to the microbiology laboratory, Division of Microbiology, Department of Clinical Pathology, Phramongkutklao Hospital. Bacterial identification was performed using matrix-assisted laser desorption/ionization time-of-flight mass spectrometry (MALDI-TOF MS; Bruker Daltonics, Bremen, Germany). CRAB was defined as *A. baumannii* exhibiting resistance to imipenem or meropenem, as determined by an automated broth microdilution system (Sensititre, Thermo Fisher Scientific, Waltham, MA, USA).

### 5.3. Procedures of Susceptible Testing

#### E-Test Method

Susceptibility testing using the E-test was performed according to the manufacturer’s instructions (Liofilchem, Roseto degli Abruzzi, Italy). Bacterial suspensions were adjusted to a 0.5 McFarland turbidity standard and inoculated onto Mueller–Hinton agar plates. E-test strips with sulbactam concentration gradients (0.016–256 µg/mL) were applied, and plates were incubated at 37 °C for 24 h. MICs were read at the point where the inhibition ellipse intersected the strip’s scale.

### 5.4. Broth Microdilution Method

BMD testing was conducted using custom-prepared microdilution panels developed by the Department of Pharmaceutical Care, Faculty of Pharmacy, Silpakorn University. Sulbactam powder (SIAM Pharmaceutical, Bangkok, Thailand) was diluted in cation adjusted Mueller–Hinton broth to final concentrations ranging from 4 to 512 µg/mL in 96-well microplates. Bacterial suspensions were standardized to 0.5 McFarland and inoculated into each well. Plates were incubated at 35 °C for 24 h. The MIC was defined as the lowest concentration at which no visible growth was observed.

### 5.5. Outcomes

The primary outcome was comparing the MIC distributions and categorical/essential agreement between the E-test and BMD methods for sulbactam. The secondary outcomes were evaluating the association between the MIC values obtained with each method and 28-day all-cause mortality in patients with CRAB pneumonia.

### 5.6. Statistical Analysis

For the primary outcome, we assessed the correlation and agreement between the E-test and BMD methods in determining sulbactam MICs. The performance of the E-test was evaluated by comparing its MIC results against the reference BMD method. For analytical purposes, any E-test MIC value that fell between twofold dilutions was rounded up to the next highest value (e.g., 24 μg/mL was recorded as 32 μg/mL).

The level of agreement was assessed using two criteria: categorical agreement (CA) and essential agreement (EA). CA was defined as the percentage of isolates (n = 134) classified in the same susceptibility category by both methods. EA was defined as the percentage of isolates for which the E-test MIC was within ±1 twofold dilution of the BMD reference MIC. Following the CLSI criteria, a method is deemed a reliable alternative if both the CA and EA exceed 90%.

Acceptable performance thresholds were defined according to CLSI guidelines: CA and EA ≥ 90%. Errors were ranked as very major error (VME: false-susceptible) and major error (ME: false-resistance) for the E-test. VME and ME of ≤1.5% and ≤3% were considered unacceptable, respectively [41]. The strength of the correlation between the MIC values obtained by the E-test and BMD was analyzed using Pearson’s correlation coefficient.

For the secondary outcome, we evaluated the association between MIC values derived from each method stratified by sulbactam dose (9 g/day vs. 12 g/day) and primary and secondary clinical outcomes of the original trial, which included 28-day mortality, discharge from hospital, discharge from intensive care unit (ICU), successful cessation of invasive mechanical ventilation, and microbiological cure by day 7. MIC values were categorized into 4 groups based on each interquartile range, as appropriate. Survival analysis was performed using Kaplan–Meier estimates, and differences between groups were assessed using the log-rank test.

Descriptive statistics were used to summarize baseline clinical and microbiological characteristics. Continuous variables are presented as mean ± standard deviation (SD) or median with interquartile range (IQR). Categorical variables are reported as frequencies and percentages. A two-tailed *p*-value < 0.05 was considered statistically significant. 

## 6. Conclusions

The sulbactam MICs among CRAB isolates differed with the testing method, with the E-test consistently overestimating the MICs compared with BMD. Only BMD-derived MICs were associated with clinical outcomes, as high-dose sulbactam improved survival exclusively in patients with very high MICs (≥128 µg/mL). These findings underscore the importance of accurate MIC testing and support BMD as the preferred method for guiding sulbactam dosing and clinical decision-making in CRAB infections. All statistical analyses were performed using R software version 4.5.1.

## Figures and Tables

**Table 1:** Differences in log_2_ dilutions of sulbactam minimal inhibitory concentrations obtained by E-test compared with broth microdilution (BMD).

Sulbactam Against Tested Isolates by BMD	Method by E-Test				No. (%) of Isolates Showing Essential Agreement (EA)
	No. (%) of Isolates Showing MIC Difference (log_2_ Dilution) of				
	−1	0	+1	≥±2	
All tested isolates (n = 134)	20	22	23	69	65 (49%)
Only isolates with MIC ≤ 64 μg/mL (n = 115)	18	22	23	52	63 (55%)
Only isolates with MIC ≤ 32 μg/mL (n = 78)	15	20	19	24	54 (69%)
Only isolates with MIC ≤ 16 μg/mL (n = 29)	0	12	11	6	23 (79%)

## Data Availability

The data presented in this study are available from the corresponding author upon reasonable request. The data are not publicly available due to ethical restrictions and patient privacy.
